# Translation rate is controlled by coupled trade-offs between site accessibility, selective RNA unfolding and sliding at upstream standby sites

**DOI:** 10.1093/nar/gkt1139

**Published:** 2013-11-14

**Authors:** Amin Espah Borujeni, Anirudh S. Channarasappa, Howard M. Salis

**Affiliations:** ^1^Department of Chemical Engineering, Penn State University, University Park, PA 16802, USA and ^2^Department of Agricultural and Biological Engineering, Penn State University, University Park, PA 16802, USA

## Abstract

The ribosome’s interactions with mRNA govern its translation rate and the effects of post-transcriptional regulation. Long, structured 5′ untranslated regions (5′ UTRs) are commonly found in bacterial mRNAs, though the physical mechanisms that determine how the ribosome binds these upstream regions remain poorly defined. Here, we systematically investigate the ribosome’s interactions with structured standby sites, upstream of Shine–Dalgarno sequences, and show that these interactions can modulate translation initiation rates by over 100-fold. We find that an mRNA’s translation initiation rate is controlled by the amount of single-stranded surface area, the partial unfolding of RNA structures to minimize the ribosome’s binding free energy penalty, the absence of cooperative binding and the potential for ribosomal sliding. We develop a biophysical model employing thermodynamic first principles and a four-parameter free energy model to accurately predict the ribosome’s translation initiation rates for 136 synthetic 5′ UTRs with large structures, diverse shapes and multiple standby site modules. The model predicts and experiments confirm that the ribosome can readily bind distant standby site modules that support high translation rates, providing a physical mechanism for observed context effects and long-range post-transcriptional regulation.

## INTRODUCTION

**Figure 1.**
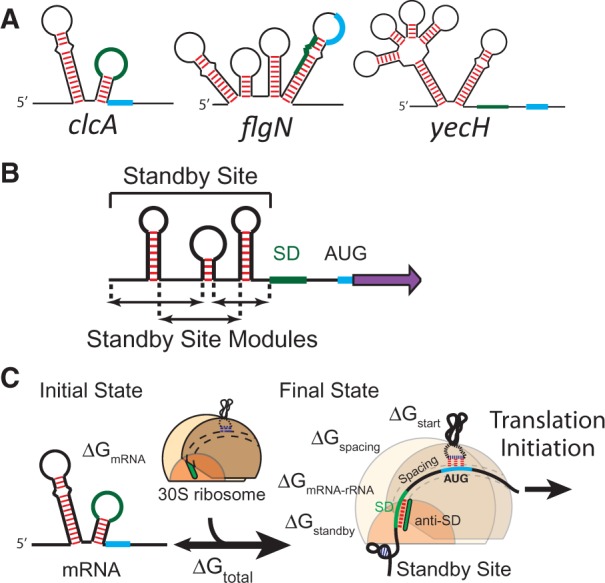
**(A)** Natural *E. coli* 5′ UTRs have diverse lengths and structures with varying binding free energy penalties to the ribosomal platform. Green and light blue regions represent SD and start codon, respectively. **(B)** 5′ UTRs are separated into multiple standby site modules, followed by a SD sequence, spacer region and a protein coding sequence. **(C)** A schematic shows the mRNA regions that contact the ribosome in its initial and final states. The ribosomal platform binds to 5′ UTRs with a binding free energy penalty Δ*G*_standby_. The sum of all binding free energies, Δ*G*_total_, determines how likely the ribosome binds to an mRNA and initiates translation.

Long 5′ untranslated regions (5′ UTRs) are commonly found in bacterial mRNAs, and their interactions with the ribosome play a central role in controlling translation initiation and post-transcriptional regulation (1–3). Changes in the 5′ UTR sequence, structure or overall shape have been found to alter an mRNA’s translation rate, including when conformational changes are triggered by the binding of proteins, small RNAs or when *cis*-acting riboswitches bind their ligand (4–8). However, our understanding of the physical mechanisms that determines the ribosome’s ability to bind long 5′ UTRs remains incomplete. While the ribosome’s interactions with Shine–Dalgarno (SD) sequences and nearby inhibitory mRNA structures have been extensively characterized (9–12) and are well-predicted by biophysical models (13–15), many natural 5′ UTRs possess further upstream sequences and structures whose interactions with the ribosome are still poorly defined (Figure 1A). Here, we use a learn-by-design approach to systematically investigate the ribosome’s interactions with long, structured 5′ UTRs, and to identify new physical mechanisms that determine the ribosome’s ability to bind and initiate translation.

According to crystal and cryo-EM structures, the 30S ribosomal complex appears to bind upstream 5′ UTRs through its platform domain, which is a positively charged surface that binds non-specifically to mRNA’s sugar phosphate backbone (3,16–18). The ribosomal platform first binds an initial landing pad, known as a ‘standby site’ (19) (Supplementary Figure S1), followed by coordinated insertion of the mRNA into the Entry channel and the 16S rRNA’s base pairing with SD like sequences to position the start codon over the P-site (18). Ribosomal S1 protein likely plays a role in remodeling mRNA structure, prior to the formation of the 30S-initiation complex, by binding to single-stranded RNA and promoting the unfolding of RNA hairpins and duplexes; however, free energy must be inputted to unfold RNA structures (20). The mRNA and ribosomal platform bind together independently of other participants, including tRNA^fMet^ and the initiation factors (1,21). Interactions that delay binding to the 5′ UTR can slow the mRNA’s translation initiation rate, including the presence of mRNA structures (17,22), competitive binding by small RNAs (23) or the absence of a standby site altogether (22). We consider a 5′ UTR as composed of one or more standby site modules, followed by a 16S rRNA binding site (a SD sequence), a spacer region and ended with a protein coding sequence (Figure 1B). An mRNA that folds into several hairpins, all located upstream of the SD sequence, will contain several standby site modules.

Thermodynamic modeling has enabled a comprehensive dissection of the ribosome’s interactions with mRNA with clearly defined contributions, quantitatively described by their free energies (Figure 1C). The most stringent test of our current understanding is to combine thermodynamic modeling with *in silico* optimization to design completely novel, non-natural RNA sequences with predicted ribosomal interactions and translation initiation rates. The differences between model predictions and experimental measurements objectively draw the boundaries between our current understanding of RNA interactions and its limitations. A growing body of evidence, from our work and others, indicates the presence of unpredicted interactions between the ribosome and long 5′ UTRs, highlighted as context effects (Supplementary Figure S1C) (14,24–28).

In this article, we decipher the biophysical rules governing the ribosome’s interactions with long, structured 5′ UTRs. We systematically alter 5′ UTR sequence, structure and shape to measure the ribosome’s interactions using *in vivo* reporter assays. We use thermodynamic modeling to quantify how the ribosome binds less accessible 5′ UTRs, accommodates or partially unfolds RNA structure, selects from multiple binding sites, slides across the mRNA and initiates translation. We develop a predictive biophysical model that combines thermodynamic minimization with a four-parameter free energy model to accurately predict the translation initiation rates of 136 long, diversely structured 5′ UTRs. Finally, we demonstrate that the ribosome can bind to distant sites and engage in long-range RNA interactions, providing a mechanism for the observed context effects and new ways for riboswitches and small RNAs to regulate translation.

## MATERIALS AND METHODS

### Strains, media and plasmid construction

Luria-Bertani (LB) media (10 g/l tryptone, 5 g/l yeast extract, 10 g/l NaCl) was obtained from BD and supplemented with 50 µg/ml chloramphenicol (Sigma-Aldrich). The expression system is derived from the pFTV1 plasmid (ColE1, Cm^R^) (14) and encodes a σ^70^ constitutive promoter (BioBrick #J23100), a synthetic 5′ UTR and a codon-optimized mRFP1 fluorescent protein. Synthetic 5′ UTRs were inserted by standard cloning techniques, utilizing either BamHI and SacI, or XbaI and SacI sites, depending on 5′ UTR length. 5′ UTRs with desired sequences were created by either annealing of complementary oligonucleotides with overhangs, or by PCR assembly of oligonucleotides with corresponding restriction sites. Plasmid variants were cloned by restriction digest of DNA backbone, purification and ligation to DNA fragments with 5′ UTR sequences and transformation of chemically competent *Escherichia coli* DH10B cells via heat shock. Correct clones were identified by DNA sequencing. All the designed 5′ UTR sequences are presented in the Supplementary Data.

### Fluorescent protein and mRNA measurements

Fluorescent protein reporter measurements were performed in 96-well format. An initial 96 deep-well plate containing 700 μl LB and 50 μg/ml chloramphenicol was inoculated, from single colonies, with up to 30 different *E. coli* DH10B cultures in an alternating pattern that excluded the outer wells. Cultures were incubated overnight at 37°C with 200 rpm orbital shaking. A fresh 96-well plate containing 200 μl LB and chloramphenicol media was inoculated by overnight cultures using a 1:100 dilution. Plates were incubated at 37°C in a plate-based spectrophotometer (TECAN M1000) with high orbital shaking. OD600 measurements were recorded every 10 min. Once a culture reached an OD600 of ∼0.15, a sample of each culture was transferred to a new plate containing 200 μl phosphate buffered saline [NaCl 137 mM, KCl 2.7 mM, Phosphate buffer 10 mM (Na_2_HPO_4_/KH_2_PO_4_ pH7.4), purchased from OmniPur] and 2 mg/ml kanamycin (Sigma Aldrich) for flow cytometry measurements. Periodic serial dilutions were repeated two more times, maintaining cultures within the exponential growth phase. Three samples were taken from each culture. The single-cell fluorescence distributions of samples were measured using a Fortessa flow cytometer (BD Biosciences). All distributions were unimodal. The autofluorescence distribution of *E. coli* DH10B cells was also measured. The arithmetic mean of each distribution was taken, and the mean autofluorescence was subtracted from each sample. The reported fluorescence values in this study are the average of three measurements (*n* = 3).

For mRNA level measurements, single colonies were first grown overnight in 5 ml LB media with chloramphenicol, then diluted to 0.01 OD600 in fresh media and grown to mid-exponential phase until reaching 1.0 OD600, as measured by a cuvette-based spectrophotometer (NanoDrop 2000C). Total RNA was extracted using the Total RNA Purification kit (Norgen Biotek). Treatment with Turbo DNAse (Ambion) eliminated genomic DNA contamination. Reverse transcription was carried out with a High Capacity cDNA Reverse Transcription kit (Applied Biosystems), followed by quantitative PCR with a TaqMan probe (5′-ACCTTCCATACGAACTTT-3′), targeting the internal coding sequence for mRFP1 (forward primer 5′-ACGTTATCAAAGAGTTCATGCGTTTC-3′ and reverse primer 5′-CGATTTCGAACTCGTGACCGTTAA-3′) and using a ABI 7300 real-time cycler. A TaqMan probe for 16S rRNA was used as an endogenous control. Triplicate measurements were each performed on two separate RNA extractions. ΔΔ*C*_t_ calculations were then averaged for each sample.

### Biophysical model calculations

The complete biophysical model employs a free energy model to calculate the total binding free energy between the ribosome and mRNA, according to the equation (Figure 1C):
(1)
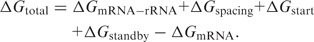



Using statistical thermodynamics, we may relate this total Gibbs free energy change to the mRNA’s translation initiation rate, *r*, according to:
(2)
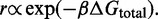



This relationship has been previously validated on 132 mRNA sequences where the Δ*G*_total_ varied from −10 to 16 kcal/mol, resulting in well-predicted translation rates that varied by over 100 000-fold (14). The apparent Boltzmann constant, *β*, has been measured as 0.45 ± 0.05 mol/kcal, which was confirmed in a second study (29).

In the initial state, the mRNA exists in a structured conformation, where its free energy of folding is Δ*G*_mRNA_ (Δ*G*_mRNA_ is negative). After assembly of the 30S ribosomal subunit, the last nine nucleotides of its 16S rRNA have hybridized to the mRNA, while all non-clashing mRNA structures are allowed to fold. The free energy of folding for this mRNA–rRNA complex is Δ*G*_mRNA–rRNA_ (Δ*G*_mRNA–rRNA_ is negative). mRNA structures that impede 16S rRNA hybridization or overlap with the ribosome footprint remain unfolded in the final state. These Gibbs free energies are calculated using a semi-empirical free energy model of RNA and RNA–RNA interactions (30,31) and the minimization algorithms available in the Vienna RNA suite version 1.8.5 (32).

Three additional interactions will alter the translation initiation rate. The tRNA^fMET^ anti-codon loop hybridizes to the start codon (Δ*G*_start_ is most negative for AUG and GUG). The 30S ribosomal subunit also prefers a five nucleotide aligned spacing (33). Non-optimal distances between the 16S rRNA binding site and the start codon lead to an energetic binding penalty. This relationship between the ribosome’s binding penalty (Δ*G*_spacing_ > 0) and aligned spacing distance was measured in our previous study (14). Finally, the 5′ UTR binds to the ribosomal platform with a free energy penalty Δ*G*_standby_, which is calculated according to Equation (5) and described in the main text. A maximally accessible standby site module will bind to the ribosomal platform with a zero Δ*G*_standby_. Gibbs free energy calculations for all mRNA sequences are listed in the Supplementary Data.

The biophysical model automatically decomposes a 5′ UTR into one or more standby site modules using the following definitions. A standby site module contains a single mRNA hairpin, surrounded by two single-stranded regions: an upstream distal binding site and a downstream proximal binding site. The lengths of the distal and proximal binding sites are denoted by *D* and *P*, respectively. The hairpin’s height, *H*, is determined by the number of nucleotides from the hairpin’s base to the mid-point of the hairpin’s loop, including bulges, internal loops and base pairs. The heights of branched hairpins are determined by averaging the branches’ heights (Supplementary Figure S3). Hairpins are only considered as components of standby site modules if they are mutually exclusive with the 16S rRNA binding site. A 5′ UTR with multiple upstream hairpins will contain multiple standby site modules. We consider a 5′ UTR without upstream hairpins to contain a single standby site module with zero hairpin height and zero distal site length. Using these definitions, the biophysical model calculates a Δ*G*_standby_ for each standby site module.

**Figure 2.**
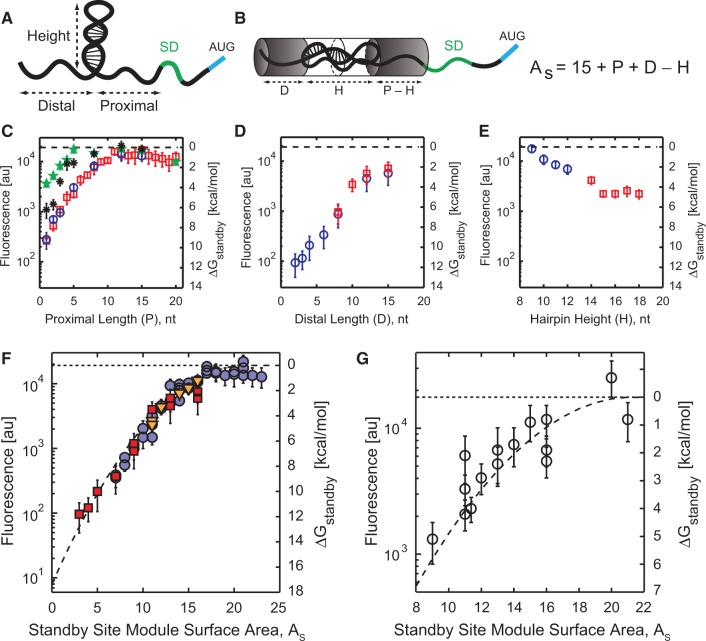
**(A)** Standby site modules are defined by a proximal and distal binding site, separated by an RNA structure. **(B)** Standby site module surface area is calculated by summing the proximal *P* and distal *D* binding site lengths, and subtracting the hairpin height *H*. The surface area is kept positive by adding a constant 15. **(C–E)** Translation rate and ribosome binding free energy penalties are measured as proximal binding site length, distal binding site length and hairpin height are increased. The Δ*G*_standby_ numbers shown in secondary *y*-axis are directly related to the data according to Equation (4). (C) Hairpin heights are 9 nt (green stars), 12 nt (black asterisks), 15 nt (red squares) or 18 nt (blue circles). (D) Hairpin heights are 15 nt (red squares) or 18 nt (blue circles). (E) Hairpin height varies from 9 to 12 nt by adding to loop length of a 6 bp stem (blue circles) or from 14 to 18 nt by adding to loop length of a 12 bp stem (red squares). **(F)** 56 characterized 5′ UTRs (*A*_s_ < 24) from parts (C–E) are combined to quantitatively relate the standby site module’s surface area to the ribosome’s translation rate and binding free energy penalty. Dashed line is a best-fit quadratic equation. **(G)** The validity of the quadratic equation is tested by measuring translation rates from 15 additional 5′ UTRs and comparing to model predictions (*R*^2 ^= 0.78, average error is 0.66 kcal/mol). Data points are the result of three measurements in 1 day. In parts (C–G), the horizontal dashed line is the translation rate and ribosome binding free energy penalty of the reference unstructured 5′ UTR.

The available single-stranded surface area, *A*_s_, of a standby site module is determined according to *A*_s_ = 15 + *P* + *D* – *H*. The key aspect of this equation is that proximal length, distal length and hairpin height have equal and interchangeable effects on the amount of single-stranded surface area. The constant 15 was selected to create a positive metric for single-stranded surface area. Based on data shown in Figure 2, increasing the hairpin height beyond 15 nt did not reduce the mRNA translation rate, regardless of the proximal or distal site lengths. Accordingly, the value of *H* in the equation is not allowed to exceed 15 nt.

The ribosomal platform’s distortion energy penalty is a quadratic function of the available RNA surface area, *A*_s_, (Figure 2), according to:
(3)
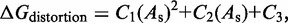

where *C*_1_, *C*_2_ and *C*_3_ are the fitted parameter values and equal to 0.038 kcal/mol/nt^2^, − 1.629 kcal/mol/nt and 17.359 kcal/mol, respectively. An alternative constant in the definition of surface area would alter these coefficients, but the overall relationship between the surface area and the distortion free energy penalty would remain the same.

### Measurements of translation rate and binding free energy penalties

In our steady-state, long-time growth conditions, translation initiation is a rate-limiting step in codon-optimized mRFP1 fluorescent protein expression; fluorescence levels are proportional to translation initiation rates under our controlled growth conditions (13), and translation initiation rate is related to total binding free energies according to a log-linear formula (Equation 2). We measure the change in apparent binding free energy penalty by comparing the fluorescence of a structured 5′ UTR with the fluorescence of a maximally accessible 5′ UTR, according to:
(4)
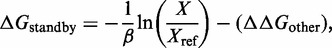

where *X* is the fluorescence from a sample synthetic 5′ UTR, *X*_ref_ is the fluorescence of the unstructured 5′ UTR, and *β* is 0.45 mol/kcal. ΔΔ*G*_other_ is the difference in free energies, between the sample and reference 5′ UTRs, that depends on other ribosomal interactions and not on the standby site. By using identical and carefully designed 16S rRNA binding sites, spacer regions and coding sequences in the 136 synthetic mRNA sequences, the value for ΔΔ*G*_other_ was zero for 123 mRNA sequences, and a maximum of 0.90 kcal/mol for the remaining sequences.

### Statistical analysis

Two-sample *T*-test, at two-tailed 5% statistical significance, was performed to compare the error distribution of different sets of model predictions. The null hypothesis assumed that the errors in two different sets of model predictions have same mean and variance. The alternative hypothesis indicates that the errors in two sets follow different distributions. The error in model prediction, ΔΔ*G*_standby_ was calculated according to the formula: ΔΔ*G*_standby_ = measured Δ*G*_standby_− predicted Δ*G*_standby_. The low probabilities (*P* < 0.05) reject the null hypothesis, showing that the differences in two sets of model predictions were statistically significant.

## RESULTS

### Standby site module surface area controls the ribosome’s translation rate

We first employed a learn-by-design approach to determine the ribosomal platform’s interactions with standby sites that contain a single hairpin, and therefore contain a single standby site module. Standby site modules are defined by their hairpin height (*H*), upstream distal binding site length (*D*) and downstream proximal binding site length (*P*) (Figure 2A; ‘Materials and Methods’ section). We designed, constructed and characterized 62 synthetic 5′ UTRs containing single standby site modules with hairpins from 9 to 18 bp long, distal binding sites from 1 to 15 nt long and proximal binding sites from 1 to 24 nt long. Long mRNA hairpins contain internal loops to avoid potential RNAse activity (34). All mRNA sequences are transcribed by the J23100 constitutive promoter, encode the mRFP1 fluorescent protein, and are designed with identical SD and spacer sequences (‘Materials and Methods’ section). Flow cytometry measurements from long-time, log-phase cultures with periodic serial dilutions revealed that mRFP1 fluorescence varied by 221-fold (Figure 2C–E). In contrast, mRNA levels varied by at most 5.8-fold, according to RT-qPCR measurements (Supplementary Figure S2). Changes in fluorescence are primarily due to changes in translation rate and, in these growth conditions, fluorescence and translation initiation rates are proportional. All sequences and measurement data are listed in the Supplementary Data.

According to the data, standby site module accessibility plays an important role in modulating the ribosome’s interactions with 5′ UTRs and controlling translation rate across a large range. In particular, lengthening the distal binding site by four nucleotides increased the mRNA’s translation rate by 4.2-fold, whereas lengthening the proximal binding site by the same amount increased it by 4.0-fold. Shortening the hairpin’s height by 4 bp increased the mRNA’s translation rate similarly by 3.1-fold. More generally, the slopes (first derivatives) of the log-transformed translation rates all have similar magnitudes (Supplementary Data, column E). Highly accessible standby site modules with long proximal binding sites or short hairpins also cause the mRNA’s translation rate to reach a plateau with the same fluorescence level as an mRNA with an unstructured 5′ UTR (Figure 2C). These observations led to the hypothesis that the geometric characteristics of the standby site module (*D*, *P*, *H*) are interchangeable, and that its available single-stranded surface area, *A*_s_, determines the ribosomal platform’s ability to bind to it.

A standby site module’s single-stranded surface area is the number of nucleotides capable of single-stranded contact with the ribosomal platform. Longer proximal or distal binding sites increase the standby site module’s surface area, while longer hairpin structures reduce the standby site module’s accessibility. mRNA structures can potentially reduce site accessibility by folding over the neighboring single-stranded sites and sequestering binding regions. A standby site module’s available surface area is calculated by summing its proximal and distal binding site lengths and subtracting the hairpin’s height (Figure 2B; ‘Materials and Methods’ section). A highly accessible standby site module has a large *A*_s_, whereas a less accessible standby site module has a small *A*_s_. We then evaluated whether the standby site module’s surface area is the key determinant by comparing *A*_s_ to the measured log-transformed translation rates. For all 62 5′ UTRs with diverse geometric features, we found a single quantitative relationship that described the effect of surface area on the mRNA’s translation rate (Figure 2F).

We now turn to thermodynamic modeling to quantify the ribosome’s interactions with standby site modules. The competitive binding of free ribosomes to the pool of intracellular mRNAs allows one to use statistical thermodynamics to relate a ribosome’s binding free energy to its translation initiation rate, according to a constant log-linear relationship (Equation 2) (13,14). We calculate the interaction energy between the ribosome and an mRNA’s standby site modules by comparing its translation to the translation rate of a reference, unstructured 5′ UTR, both with an identical SD sequence, spacer region and protein coding sequence (Equation 4). Using the data in Figure 2C–E, we found that the standby site binding free energy penalty, ΔG_standby_, varied from 0 to 11.8 kcal/mol when changing its geometric characteristics, and that a simple quadratic function succinctly described the effect of site accessibility on the ribosome’s binding free energy penalty (R^2^ = 0.97, average error is 0.51 kcal/mol) (Figure 2F). A maximally accessible standby site module has a zero binding free energy penalty (ΔG_standby_ = 0), indicating that the mRNA’s translation initiation rate is not reduced by the ribosomal platform’s interactions. A standby site module with a positive binding free energy penalty (ΔG_standby_ > 0) has reduced the mRNA’s translation initiation rate by decreasing the ribosomal platform’s ability to initially bind the mRNA.

Next, we critically tested the parameterized model by constructing 15 new 5′ UTRs and measuring their translation initiation rates and ribosome binding free energy penalties. The 5′ UTRs each contain a standby site module with diverse structures, between 43 and 91 nt long, including variable-length distal and proximal binding sites, hairpin heights, and including two- and three-branched structures (Supplementary Data). For branched structures, we show that the relevant hairpin height is the average across the multiple branches (Supplementary Figure S3) (see also Supplementary Text). The quadratic relationship accurately predicted the ribosome’s translation rate and binding free energy penalty (*R*^2 ^= 0.78, average error is 0.66 kcal/mol), confirming that standby site module’s accessibility controls the ribosome’s ability to bind and initiate translation (Figure 2G). The quadratic relationship contains three parameters, which are now treated as constants (Equation 3; ‘Materials and Methods’ section).

### Coupled energetic trade-offs when unfolding structures in standby site modules

Prior to initiating translation, the 30S ribosome does not have an external source of energy. Initiation factor 2 does not hydrolyze GTP until after translation has been initiated, during 50S recruitment (22). Consequently, the 30S complex expends its own free energy to unfold mRNA structures. Previous studies have shown that mRNA structures that sequester the 16S rRNA binding site are unfolded prior to initiating translation (9,14,22). The mRNA’s translation initiation rate is reduced according to the free energy penalty for unfolding these structures, as described in Equations (1) and (2) (‘Materials and Methods’ section). However, it remains unclear whether mRNA structures located upstream of SD sequences, within standby site modules, are unfolded or accommodated. We next investigated how the ribosome unfolds hairpins in long 5′ UTRs.

By definition, there is a coupled thermodynamic relationship between the unfolding of an mRNA hairpin and increasing the accessibility of the corresponding standby site module, where the accessibility of standby site module controls the ribosomal platform’s distortion energy penalty Δ*G*_distortion_ (see ‘Materials and Methods’ section). Unfolding a single base pair in a module’s hairpin will increase the standby site module’s surface area by three nucleotides, the hairpin’s height decreases by one and the lengths of both the proximal and distal binding sites increase by one. Therefore, unfolding a module’s hairpin will lead to higher accessibility, a lower Δ*G*_distortion_ and a higher translation rate. However, unfolding base pairs requires an input of free energy (Δ*G*_unfolding_ > 0), which will lead to a lower translation rate. Consequently, there is an energetic trade-off between unfolding mRNA structures, and increasing the surface accessibility of standby site modules. The ribosome may completely unfold a structured standby site module to maximize its surface area, but the energetic cost may be high (high Δ*G*_unfolding_ penalty with Δ*G*_distortion_ = 0). In contrast, the ribosome may fully accommodate a standby site module’s hairpin, without unfolding it, even though its low surface accessibility will penalize the ribosome’s ability to bind to it (high Δ*G*_distortion_ penalty with Δ*G*_unfolding_ = 0).

**Figure 3.**
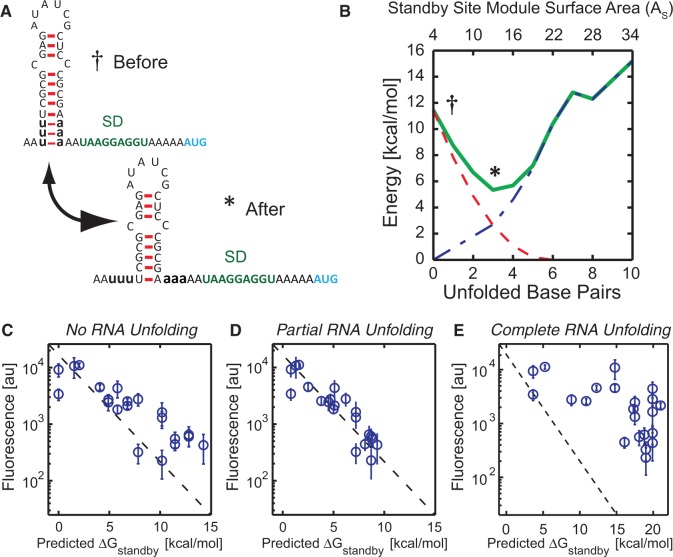
**(A** and **B)** Competition between ribosomal distortion and selective RNA unfolding is illustrated on a structured 5′ UTR with a weak hairpin. The ribosome’s distortion penalty Δ*G*_distortion_ (dashed red line), hairpin unfolding penalty Δ*G*_unfolding_ (dotted dashed blue line) and their sum Δ*G*_standby_ (green solid line) are shown as the hairpin’s closing base pairs are unfolded. The total binding free energy penalty has a local minima when three base pairs are unfolded. See also Supplementary Figure S4. **(C–E)** Scatter plots comparing measured translation rates from 22 synthetic 5′ UTRs and model predictions to test three mechanisms for the ribosome’s unfolding of RNA structures. The average difference between model predictions and measurements is (C) 2.42 kcal/mol when not unfolding structures, (D) 0.90 kcal/mol when partially unfolding structures and (E) 10.24 kcal/mol when fully unfolding structures. Comparative *P*-values from two-sample *T*-tests are 0.004 (C versus D) and 10^−13^ (D versus E). Data points are the result of three measurements in 1 day. In parts (C–E), the diagonal dashed line is the predicted translation rate according to Equation (2).

Based on thermodynamic principles, we hypothesize that the ribosome will ‘selectively’ and ‘partially’ unfold structured standby site modules to minimize its total binding free energy penalty (Δ*G*_standby_ = Δ*G*_distortion_ + Δ*G*_unfolding_). Figure 3A and B presents an illustrative example of this principle and our use of minimization to calculate the ribosome’s binding free energy penalty to a standby site module. A 5′ UTR with a single standby site module initially contains a short proximal binding site (*P* = 2 nt), a short distal binding site (*D* = 2 nt) and a long hairpin (*H* = 15 nt), resulting in a largely inaccessible 5′ UTR (*A*_s_ = 4). Based on the standby site module’s surface area, the ribosome has an initial distortion energy penalty of 11.5 kcal/mol. However, the ribosome can selectively unfold the hairpin’s closing A:U base pairs, increasing the standby site module’s surface area and lowering its Δ*G*_distortion_ at the expense of increasing its Δ*G*_unfolding_. Unfolding 1 bp increases the standby site module’s surface area by 3 nt, decreases the distortion energy penalty to 7.8 kcal/mol and increases the unfolding energy penalty to 0.90 kcal/mol, leading to a total binding free energy penalty of 8.7 kcal/mol. The ribosome can continue to unfold base pairs to minimize its total binding free energy penalty; unfolding 3 bp at a cost of 2.7 kcal/mol leads to a distortion energy penalty of 2.6 kcal/mol, and a total binding free energy penalty of 5.3 kcal/mol (Figure 3B). Two additional examples are shown in Supplementary Figure S4. The unfolding free energies are calculated using the Vienna RNA suite with the Turner 1999 RNA parameters (see ‘Materials and Methods’ section). Importantly, thermodynamic minimization does not add any new parameters to the model.

We tested the proposed mechanism by characterizing the translation rate of 22 synthetic 5′ UTRs (Figure 3C–E) that contain single standby site modules with varied geometries (*D* = 0 to 7 nt and *P* = 1 to 20 nt) and differing hairpin sequences, sizes and folding energetics (Supplementary Figure S5A). We employed the minimization principle to calculate the number of unfolded base pairs and to predict the ribosome’s binding free energy penalty, Δ*G*_standby_ (Figure 3D). As comparisons, we performed the same calculation while asserting that either no additional energy was inputted to unfold hairpins (high Δ*G*_distortion_ and Δ*G*_unfolding_ = 0) (Figure 3C) or that hairpins were completely unfolded by the ribosome (high Δ*G*_unfolding_ and Δ*G*_distortion_ = 0) (Figure 3E).

The data supports our hypothesis that the ribosome partially and selectively unfolds structured standby site modules to minimize its total binding free energy penalty (Δ*G*_standby_), rather than separately minimize its distortion or unfolding penalties (average error is 0.90 kcal/mol when minimizing Δ*G*_standby_, 2.42 kcal/mol when Δ*G*_unfolding_ = 0 and 10.24 kcal/mol when Δ*G*_distortion_ = 0; comparative *P*-values are 0.004 and 10 ^−^^13^, respectively). Remarkably, in all 22 cases, only 1–3 bp of each hairpin are predicted by the model to be unfolded, while accommodating the remaining large RNA structures.

### Non-cooperative binding to multiple standby site modules

**Figure 4.**
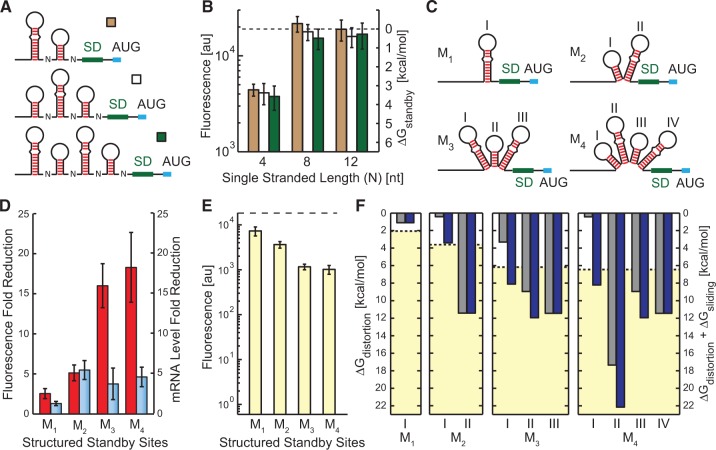
**(A)** Synthetic 5′ UTRs with multiple, evenly spaced standby site modules. Spacing *N* is varied from 4 to 12 nt. **(B)** Measured translation rates are compared to standby site module multiplicity, showing the absence of cooperative binding to multiple standby site modules, regardless of limiting surface areas. The Δ*G*_standby_ numbers shown in secondary *y*-axis are directly related to the data according to Equation (4). **(C)** Synthetic 5′ UTRs with one upstream and multiple internal standby site modules, labeled with roman numbers. **(D)** The reduction in fluorescence (red bars) and mRNA levels (blue bars) of 5′ UTRs M_1_ to M_4_, compared to an unstructured 5′ UTR (see also Supplementary Figure S2). **(E)** Fluorescence measurements of 5′ UTRs M_1_ to M_4_ show that upstream standby site modules can support high translation rates. **(F)** Model predictions are shown for 5′ UTRs M_1_ to M_4_ using either Δ*G*_distortion_ (gray bars) or Δ*G*_distortion_ + Δ*G*_sliding_ (blue bars) for each standby site module (I–IV, corresponding to labels in part C), compared to the ribosome’s apparent binding free energy penalties (dashed lines, yellow region) (average error with and without the sliding penalty is 1.15 and 3.24 kcal/mol, respectively; *P*-value is 0.013). Δ*G*_distortion_ for each standby site module was calculated according to Equation (3). In parts B and E, the horizontal dashed line is the translation rate and ribosome binding free energy penalty of the unstructured 5′ UTR.

We have shown that the ribosome can accommodate large structures of standby site modules and engage in frequent translation, but it is unclear how the presence of multiple standby site modules in a 5′ UTR will affect its translation rate. The presence of multiple standby site modules could allow the ribosome to cooperatively bind mRNA and increase its translation rate. We next characterized nine synthetic 5′ UTRs (76–164 nt long) with up to four standby site modules to investigate the potential for cooperative binding (Figure 4A). If the ribosome can engage in cooperative binding, then the additional standby site modules will increase the overall available surface area, reduce the ribosome’s binding free energy penalty and increase the translation initiation rate. In particular, we expect the greatest cooperativity when the available surface area of a single standby site module is limited.

We found that 5′ UTRs with two, three or four standby site modules were all translated at the same rate, indicating an absence of cooperative binding (Figure 4B), including when the available surface areas for individual standby site modules were significantly lowered. Therefore, even when the combined surface areas of multiple standby site modules could potentially increase contact with the ribosomal platform and increase binding, a cooperative increase in translation rate was not observed. Consequently, the data supports a mechanism whereby the ribosome binds to only a single standby site module prior to initiating translation.

### Distant standby site modules can support high translation rates

We next investigated whether any standby site module could be bound by the ribosome, even if located far upstream of the start codon. Using the model’s predictions, we forward-engineered multi-standby site module synthetic 5′ UTRs where the most distantly upstream site is the only one capable of supporting high translation rates (Figure 4C). Internal standby site modules have long hairpins and limited surface areas (model predictions: Δ*G*_standby_ between 9.0 and 17.4 kcal/mol) compared to the maximally accessible upstream standby site modules. Therefore, if the ribosome binds the farthest upstream standby site module, its translation rate will be at least 12.7-fold higher than binding to the internal standby site modules. We measured fluorescence levels with flow cytometry and mRNA levels with RT-qPCR. Compared to an unstructured 5′ UTR, the mRNA levels decreased by only 1.3- to 5.5-fold, while the fluorescence levels were correspondingly reduced between 2.5- and 18.3-fold, showing that changes in fluorescence were mainly due to changes in translation rate and ribosome binding free energy penalties (Figure 4D).

We found that all the mRNAs’ translation rates remained very high, even when the accessible standby site module was located over 100 nt upstream of the start codon (M4 5′ UTR) (Figure 4E). In particular, the M2 5′ UTR is translated at a 33.8-fold higher rate when compared to the translation rate that could be supported by binding to the standby site module closest to the start codon. Moving the accessible standby site module upstream, away from the start codon, does not significantly reduce the translation rate. Based on these measurements, the apparent standby site binding penalties are 3.6, 6.2 and 6.5 kcal/mol for M2 to M4 5′ UTRs (Figure 4F), respectively, which is much lower than the internal standby site modules’ predicted binding free energy penalties (9.0–17.4 kcal/mol). These measurements suggest that the ribosome will bind to the standby site module that has the lowest binding free energy penalty, regardless of its distance from the start codon.

### The ribosomal platform can slide across upstream structures

Several mechanisms would allow the ribosomal platform to bind distant, upstream standby site modules and transition to a pre-initiation complex at the start codon. After the ribosome has bound to the upstream standby site module, the 30S complex could engage in diffusive hopping that would increase its local concentration and assembly rate at the start codon. The rate of assembly would largely depend on the physical distance between the standby site module and start codon, and not on the presence of RNA structures. Alternatively, the ribosome could employ a full contact, sliding mechanism that reorients the mRNA until a stable pre-initiation complex has formed. The positively charged surface of the ribosomal platform makes it ideal for maintaining contact with a 5′ UTR until 16S rRNA hybridization has anchored the mRNA. Unlike a diffusive hopping mechanism, the presence of mRNA structures would impede this sliding motion and reduce the pre-initiation complex’s assembly rate.

Further analysis of our measurements shows that RNA structures in between the upstream standby site module and start codon partly attenuate the mRNA’s translation rate (Figure 4E). The apparent binding free energy penalty of the M2 5′ UTR (3.6 kcal/mol) is higher than the upstream standby site module’s predicted binding free energy penalty (0.4 kcal/mol). This energetic difference cannot be explained by binding to the internal standby site module (11.4 kcal/mol), or by the unfolding of the downstream RNA structure (17.5 kcal/mol). Thus, the free energy difference (3.6 – 0.4 = 3.2 kcal/mol) is significant, but much lower than predicted by alternative binding to other standby site modules or from unfolding of RNA structures.

Our data supports the presence of a sliding mechanism for the ribosomal platform. Intervening mRNA structures are not unfolded, but are pushed aside with an energetic cost. Using our measurements of the M2 5′ UTR, we estimate that 3.2 ± 0.5 kcal/mol free energy is required to push aside the single RNA structure with a height of 15 nt, yielding a coefficient of 0.20 ± 0.04 kcal/mol per hairpin height. We then determined whether including this sliding penalty could improve the model’s ability to predict the translation rate of long 5′ UTRs with multiple standby site modules with ‘hairpins of differing heights’. For each standby site module, we calculate the heights of all downstream RNA structures, and multiplied the sum by the coefficient (*c* = 0.2 kcal/mol/nt) to yield a free energy penalty of sliding, Δ*G*_sliding_. The sliding free energy penalties were 0, 3.0, 4.8 and 7.8 kcal/mol for M1 to M4 5′ UTRs, respectively. We add together the sliding, distortion and unfolding free energy penalties to calculate the ribosome’s binding free energy penalty to each standby site module.

Figure 4E shows the measured translation initiation rates for M1 to M4, alongside the predicted binding free energy penalties (Figure 4F), both with and without the sliding free energy penalty. The data shown in Figure 4E and F are on the same scale, according to the log-linear relationship between translation initiation rate and binding free energy differences (‘Materials and Methods’ section), enabling a comparison between the measurements and the two predictions. The introduction of the sliding free energy penalty enabled the biophysical model to more accurately predict the translation rate from longer 5′ UTRs with several standby site modules (average error is 1.86 kcal/mol with the sliding energy penalty, compared to 4.45 kcal/mol without the sliding energy penalty; *P*-value is 0.013).

### A biophysical model predicts translation rates from diversely structured 5′ UTRs

Altogether, the ribosome binds to 5′ UTRs by selecting the most geometrically accessible standby site module that requires the least amount of RNA unfolding. Standby site module selection is not limited by distance to the start codon, and upstream standby site modules can support high rates of translation. We identified that ribosomal distortion, selective RNA unfolding and ribosomal sliding control standby site module selection and binding energetics, and combine them together to create a single biophysical model. The ribosome’s binding free energy penalty to the standby site modules in a structured 5′ UTR is calculated according to
(5)
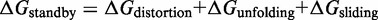



All three Gibbs free energies are positive, and a long, unstructured 5′ UTR would bind to the ribosomal platform without an energetic penalty (Δ*G*_standby_ = 0 kcal/mol). All RNA energetics are calculated using a semi-empirical free energy model of RNA and RNA–RNA interactions (30,31) and the minimization algorithms available in the Vienna RNA suite version 1.8.5 (32). This model extends our previous biophysical model that focused on downstream mRNA interactions, including the 16S rRNA binding site, spacer region and downstream RNA structures (13,14).

**Figure 5.**
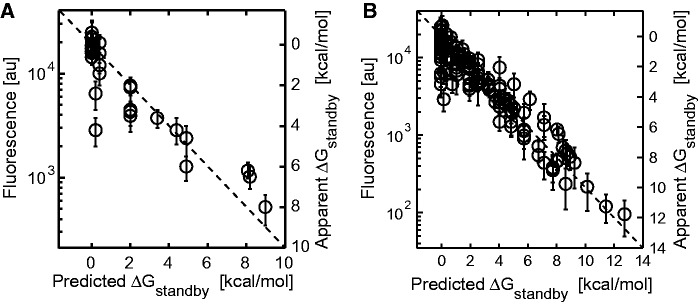
**(A)** A scatter plot showing fluorescence measurements and apparent Δ*G*_standby_ energy penalties compared to predicted Δ*G*_standby_ energy penalties for 28 synthetic 5′ UTRs with diverse structures (average error is 0.79 kcal/mol and *R*^2 ^= 0.83). (**B**) The same comparison for the 136 synthetic 5′ UTRs characterized in this study (average error is 0.75 kcal/mol and *R*^2 ^= 0.89). In parts (A and B), the apparent Δ*G*_standby_ numbers shown in secondary *y*-axis are directly related to the data according to Equation (4). An *x = y* diagonal line is shown (dashed).

We further tested the accuracy of the biophysical model by characterizing an additional 28 diversely structured 5′ UTRs (68–164 nt long) with two to four standby site modules of varying geometries, RNA structure energetics and hairpin heights (Supplementary Figure S5B). The biophysical model was able to accurately predict the relative translation rates and ribosome binding free energy penalties (average error is 0.79 kcal/mol and *R*^2 ^= 0.83) (Figure 5A). Overall, the biophysical model contains four, empirically determined parameter values, but can accurately predict the binding free energy penalties and translation rates of the 136 synthetic 5′ UTRs characterized in this study (average error is 0.75 kcal/mol and *R*^2^ = 0.89) (Figure 5B).

### Genome-wide predictions of 5′ UTR translation rates

We next employed the biophysical model to examine how standby sites in genomic 5′ UTRs control their translation initiation rates. From the annotated *E. coli* MG1655 genome, provided by EcoCyc (35), we collected 3430 5′ UTRs that varied from short leaders to gene-sized regulatory hot spots with average 5′ UTR length of 100 nt. The 5′ UTR sequences from the +1 of the mRNA transcript to +100 after the protein coding sequence’s start codon were inputted into the biophysical model. For individual 5′ UTRs, a list of standby site modules was automatically generated. For each standby site module, the surface area *A*_s_, distortion energy penalty Δ*G*_distortion_, unfolding energy penalty Δ*G*_unfolding_ and sliding energy penalty Δ*G*_sliding_ were calculated. These calculations are combined to determine the Δ*G*_standby_ for each standby site module. Because of the absence of cooperative binding, as shown, the standby site module with the lowest binding free energy penalty is selected as the one controlling the ribosome’s translation initiation rate.

**Figure 6.**
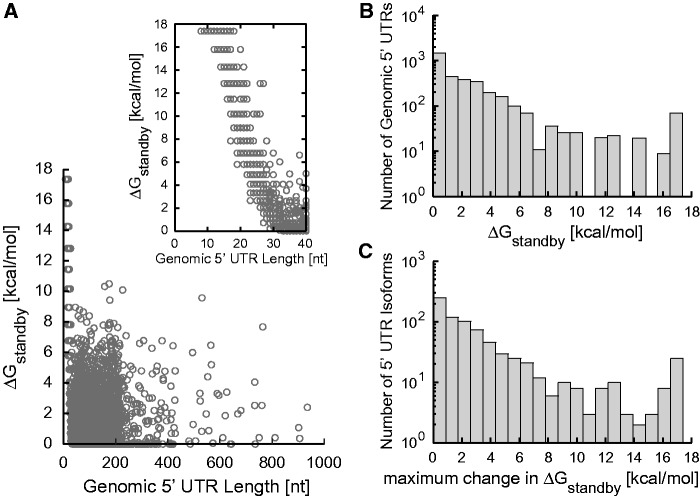
**(A)** The ribosomal platform binding free energy penalties, Δ*G*_standby,_ for 3430 transcribed 5′ UTRs from the *E. coli* MG1655 genome (EcoCyc release 17.1) are compared to their lengths. The inset shows the Δ*G*_standby_ of short 5′ UTRs. **(B)** The number of genomic 5′ UTRs with different values of Δ*G*_standby_ is shown. **(C)** 5′ UTR isoforms within transcripts across the genome affect their standby site module characteristics, as quantified by the maximum calculated change in Δ*G*_standby_.

According to the model calculations, we found that the lengths of the natural 5′ UTRs do not correlate with their ability to bind the ribosomal platform (Figure 6A). Short 5′ UTRs with low accessibility can inhibit translation equally well, compared to long 5′ UTRs where accessibility is limited by RNA structures. The model calculations indicate that the ribosomal platform can bind to natural 5′ UTRs with a wide range of energetic penalties (up to 17.4 kcal/mol), repressing translation by up to 2500-fold (Figure 6B). Long 5′ UTRs with low Δ*G*_standby_ can be subjected to translation repression when trans-acting small RNAs bind and reduce their single-stranded accessibility (23,29,36–38), or when *cis*-acting riboswitches reduce accessibility when bound to a chemical ligand (39–43). In one example, the standby site in the *tisB* 5′ UTR is occluded when bound by the IstR-1 small RNA, and its translation is repressed (23). In contrast, long 5′ UTRs with high Δ*G*_standby_ can be targets for translation activation when structural remodeling by small RNAs or riboswitches increases their surface accessibility. RprA small RNA has been shown to bind and up-regulate the translation of *rpoS* mRNA with the help of Hfq-protein (44).

The model predicts that both types of translation regulation occur in natural 5′ UTRs. In particular, 39% of genomic 5′ UTRs have standby site modules whose distal or proximal binding sites are longer than 15 nt, which are potent targets for translational repression; in contrast, 9% of 5′ UTRs contain standby site modules with low accessibilities (*A*_s_ < 10), making them targets for translation activation. Moreover, 79 diverse 5′ UTRs with an average length of 175 nt have positive sliding energy penalties (Δ*G*_sliding_), indicating that distant standby site modules are controlling translation, and making them potential targets for long-range regulation.

Further, there are 769 operons where variability in their transcriptional start sites, due to adjacent or overlapping promoters, result in 5′ UTRs with different lengths and mRNA structures. For example, the *mmuPM* operon has five different transcriptional start sites, leading to 5′ UTR isoforms with lengths from 9 to 205 nt. The biophysical model predicts a 1088-fold change in the translation initiation rate of *mmuP*, as a result of changes in the structure and surface area of the different standby site modules. Overall, across all 5′ UTR isoforms within the genome, model calculations show large changes in translation initiation rates through alterations in the standby site modules’ characteristics (Figure 6C). These differences imply that switching promoter usage, due to changing transcription factor or sigma factor levels, can modulate an mRNA’s translation rate, and potentially create standby site modules whose accessibility can be additionally regulated by translation factors. However, further investigation is necessary to examine the 5′ UTRs of isoforms and the regulation of their translation rates.

### Biophysical modeling to calculate translation rates from split ribosome binding sites

**Figure 7.**
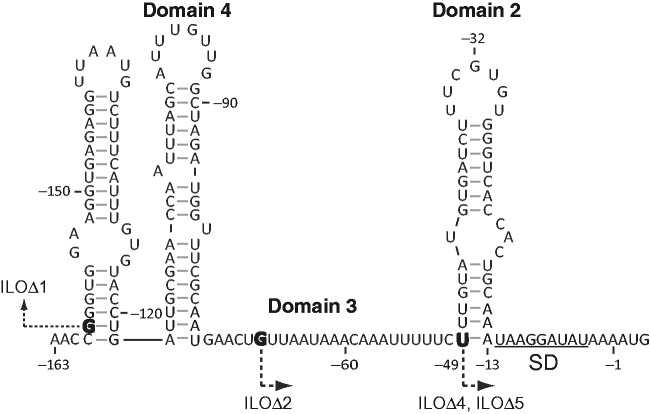
The sequence and structure of *E. coli* thrS 5′ UTR are shown. Similar to Sacerdot *et al.* (45), nucleotide numbering is negative for the upstream 5′ UTR with respect to the start codon. The list of mutations is: BS4-9: G(-32)>A; BS4-9/CS29: G(-32)>A and G(-46)>A; L19: A(-13)>C, L7: U(-49)>G; CS30Δ2: deletion of domain 2 from A(-14) to U(-48); ILOΔ1: transcription begins at G(-159); ILOΔ2: transcription begins at G(-68); ILOΔ4: transcription begins at G(-49), U(-49)>G and A(-13)>C; ILOΔ5: transcription begins at G(-49), U(-49)>G and G(-46)>A. Bolded nucleotides are the positions of new transcriptional start sites.

Sacerdot *et al.* (45) studied the translation of the 5′ UTR *of E. coli* thrS mRNA that uses a split ribosome binding site. The biophysical model classifies the 5′ UTR as containing three standby site modules with large RNA structures and varying distal and proximal binding site lengths (Figure 7). The authors concluded that a 24 nt long single stranded region (domain 3) is essential for ribosome binding and that the ribosome can accommodate a long hairpin (domain 2) without unfolding it, which was later supported by crystallographic data (3). The biophysical model’s calculations are strongly consistent with their conclusions. In particular, a mutation that deleted the entire domain 3 and its upstream region (ILOΔ4) repressed the translation rate by over 50-fold, where it significantly reduced the standby site accessibility (from *A*_s_ = 24 and Δ*G*_distortion_ = 0 to *A*_s_ = 0 and Δ*G*_distortion_ = 17.4 kcal/mol). To offset this large energetic burden, model calculations indicate that the ribosomal platform partially unfolded 6 bp from the domain 2 hairpin, which minimized its binding free energy penalty to a Δ*G*_standby_ of 5.32 kcal/mol (*A*_s_ = 20.5, Δ*G*_distortion_ = 0.02 and Δ*G*_unfolding_ = 5.3 kcal/mol). Destabilization of the domain 2 hairpin (ILOΔ5) restored the mRNA’s translation to its wild-type rate by increasing the surface area of the standby site to 20.5 nt, resulting in an almost zero Δ*G*_standby_. The biophysical model calculations indicate that other mutations to the *thrS* 5′ UTR (L6, M1, N1, BS4-9, BS4-9/CS29, L19, L7, CS30Δ2, ILOΔ1 and ILOΔ2; see Figure 7) did not alter its standby site module surface area or RNA structure; the maximum observed change in translation was 2.5-fold for these mutations.

## DISCUSSION

We designed and characterized synthetic mRNA sequences and applied biophysical modeling to decipher the rules for the ribosome’s interactions at structured 5′ UTRs. The 30S ribosome searches for a single standby site module that supports the highest translation rate, regardless of its distance from the start codon. The ribosome’s binding free energy penalty to individual standby site modules is governed by a competing trade-off between the accessibility of standby site modules (Figure 2) and the unfolding of RNA structures to increase accessibility (Figure 3). We demonstrate that thermodynamic minimization predicts the extent of the ribosome’s selective and partial unfolding of RNA structures. Our data supports a sliding mechanism whereby downstream RNA structures are not unfolded, but attenuate translation rate in a height-dependent manner (Figure 4).

Importantly, initially designed sequences have well-defined features that perturb the ribosome’s individual interactions with mRNA, allowing us to precisely measure their effect on the ribosome’s binding free energy penalty. The forward design of synthetic sequences, according to model predictions, quantitatively tests our knowledge of these interactions and the model’s ability to predict their strengths. Overall, the biophysical model employs first-principles calculations with four empirically measured constants to accurately predict the translation initiation rates of 136 mRNAs with diversely structured, long 5′ UTRs (Figure 5).

Based on our findings, there is also the potential for long-range regulation of translation. Long, structured 5′ UTRs can feature several upstream standby site modules extending over 100 nt away from an open reading frame. Any of these standby site modules are potential binding sites for the ribosome, according to their binding free energy penalties, but only a single standby site module is ultimately bound. Both *cis*-acting and *trans*-acting factors can modulate individual standby site module surface accessibilities to control standby site module selection and the resulting translation rate (3,17,23). Importantly, the regulation of standby site module selection will produce discrete step changes in translation rate that can further regulate downstream processes according to non-Boolean, multi-state logic.

In this study, we introduce a new metric to quantify the surface area of a standby site module, which was validated using diverse, but canonical, RNA secondary structures. The metric relates the geometric characteristics of the standby site module (*P*, *D*, *H*) to its available single-stranded surface area, implicitly using coefficients of one to indicate interchangeability. Additional measurements will be needed to precisely measure these coefficients, and relate them to the helical shape of A-form RNA. In addition, tertiary structures, such as G-quadruplexes and pseudoknots, will likely have a differently defined metric of surface area. Further investigation will be necessary to determine the effect of tertiary RNA structures on standby site accessibility, ribosome binding and translation initiation.

As suggested by the biophysical model, the ribosomal platform’s ability to bind standby site modules with low surface area relies on its structural flexibility. Previously published cryo-EM data shows that the platform domain in the 30S subunit, in both the free and 50S-bound states, can occupy variable conformational states (46). The two halves of the platform, which are stabilized by ribosomal proteins at its surface, switch conformations during translation (47). Restraining this flexibility will reduce the ribosome’s translation initiation rate; for example, when the antibiotic Edeine binds 16S ribosomal RNA helices H23 and H24 (48). Consequently, the ribosomal platform’s structural flexibility appears to be an essential aspect of translating mRNAs with structured 5′ UTRs.

Similarly, the rigidity of single-stranded RNA likely plays an important role in modulating the ribosome’s distortion penalty, due to the entropic differences of binding flexible or rigid macromolecules (49). Similar to DNA, polyA RNA sequences are less flexible than mixed or polyU RNA sequences (50,51). Differences in RNA rigidity have been shown to alter the ribosome’s binding affinity to spacer regions separating the SD and start codon sequences (52); binding free energy differences are ∼2.5 kcal/mol between polyA and polyAU spacer sequences, and ∼5 kcal/mol between polyA and polyU spacer sequences. Incorporating a sequence-dependent model for RNA rigidity into the distortion penalty calculations could potentially increase their accuracy.

Analysis of the model’s error can lead to further insight into the ribosome’s interactions. The model’s predictions have a normally distributed error for 90% of sequences (Supplementary Figure S6). Thirteen outliers have errors between 2 and 4 kcal/mol (Supplementary Data). Some outliers are due to the effects of increased mRNA degradation of 5′ UTRs with very long proximal or distal binding sites (*P*, *D* > 20 nt) (Supplementary Figure S2). In others, extremely short proximal or distal binding sites can result in non-canonical base pairings that alter the ribosome’s binding affinity. For example, in the absence of a proximal binding site (*P* = 0 nt), co-axial stacking will take place between the nucleotides in the distal binding site and the mRNA–rRNA duplex that anchors the ribosome to the mRNA. Similarly, co-axial stacking will play a larger role in predicting the effects of small RNAs that bind to sites nearby RNA structures.

As the list of interactions that control gene expression continues to grow, we will stretch the limits of human pattern recognition and the use of observational correlations. Biophysical modeling offers a comprehensive approach to account for all known interactions with the gene expression machinery, identify gaps, design experiments, precisely measure strengths and make testable predictions. The accuracy of these models will go hand-in-hand with our ability to engineer genetic systems without trial-and-error. We have incorporated the ribosome’s interactions with structured standby site modules into an improved biophysical model, called the RBS Calculator v2.0. A software implementation and user-friendly web interface is available at http://salis.psu.edu/software.

## SUPPLEMENTARY DATA

Supplementary Data are available at NAR Online.

Supplementary Data

## References

[1] Tsai A, Petrov A, Marshall RA, Korlach J, Uemura S, Puglisi JD (2012). Heterogeneous pathways and timing of factor departure during translation initiation. Nature.

[2] Simonetti A, Marzi S, Jenner L, Myasnikov A, Romby P, Yusupova G, Klaholz BP, Yusupov M (2009). A structural view of translation initiation in bacteria. Cell. Mol. Life Sci..

[3] Jenner L, Romby P, Rees B, Schulze-Briese C, Springer M, Ehresmann C, Ehresmann B, Moras D, Yusupova G, Yusupov M (2005). Translational operator of mRNA on the ribosome: how repressor proteins exclude ribosome binding. Science.

[4] Beisel CL, Updegrove TB, Janson BJ, Storz G (2012). Multiple factors dictate target selection by Hfq-binding small RNAs. EMBO J..

[5] Storz G, Vogel J, Wassarman KM (2011). Regulation by small RNAs in bacteria: expanding frontiers. Mol. Cell.

[6] Bastet L, Dubé A, Massé E, Lafontaine DA (2011). New insights into riboswitch regulation mechanisms. Mol. Microbiol..

[7] Beisel CL, Smolke CD (2009). Design principles for riboswitch function. PLoS Comput. Biol..

[8] Lease RA, Belfort M (2000). A trans-acting RNA as a control switch in *Escherichia coli*: DsrA modulates function by forming alternative structures. Proc. Natl Acad. Sci. U.S.A..

[9] De Smit MH, Van Duin J (1994). Control of translation by mRNA secondary structure in *Escherichia coli*. A quantitative analysis of literature data. J. Mol. Biol..

[10] De Smit MH, Van Duin J (1994). Translational initiation on structured messengers. Another role for the Shine-Dalgarno interaction. J. Mol. Biol..

[11] Osterman IA, Evfratov SA, Sergiev PV, Dontsova OA (2013). Comparison of mRNA features affecting translation initiation and reinitiation. Nucleic Acids Res..

[12] Bugaut A, Balasubramanian S (2012). 5′-UTR RNA G-quadruplexes: translation regulation and targeting. Nucleic Acids Res..

[13] Salis HM (2011). The ribosome binding site calculator. Methods Enzymol..

[14] Salis HM, Mirsky EA, Voigt CA (2009). Automated design of synthetic ribosome binding sites to control protein expression. Nat. Biotechnol..

[15] Carothers JM, Goler JA, Juminaga D, Keasling JD (2011). Model-driven engineering of RNA devices to quantitatively program gene expression. Science.

[16] Simonetti A, Marzi S, Myasnikov AG, Fabbretti A, Yusupov M, Gualerzi CO, Klaholz BP (2008). Structure of the 30S translation initiation complex. Nature.

[17] Marzi S, Myasnikov AG, Serganov A, Ehresmann C, Romby P, Yusupov M, Klaholz BP (2007). Structured mRNAs regulate translation initiation by binding to the platform of the ribosome. Cell.

[18] Yusupova GZ, Yusupov MM, Cate J, Noller HF (2001). The path of messenger RNA through the ribosome. Cell.

[19] De Smit MH, Van Duin J (2003). Translational standby sites: how ribosomes may deal with the rapid folding kinetics of mRNA. J. Mol. Biol..

[20] Qu X, Lancaster L, Noller HF, Bustamante C, Tinoco I (2012). Ribosomal protein S1 unwinds double-stranded RNA in multiple steps. Proc. Natl Acad. Sci. U.S.A..

[21] Milón P, Maracci C, Filonava L, Gualerzi CO, Rodnina MV (2012). Real-time assembly landscape of bacterial 30S translation initiation complex. Nat. Struct. Mol. Biol..

[22] Studer SM, Joseph S (2006). Unfolding of mRNA secondary structure by the bacterial translation initiation complex. Mol. Cell.

[23] Darfeuille F, Unoson C, Vogel J, Wagner EGH (2007). An antisense RNA inhibits translation by competing with standby ribosomes. Mol. Cell.

[24] Mutalik VK, Guimaraes JC, Cambray G, Mai Q-A, Christoffersen MJ, Martin L, Yu A, Lam C, Rodriguez C, Bennett G (2013). Quantitative estimation of activity and quality for collections of functional genetic elements. Nat. Methods.

[25] Cardinale S, Arkin AP (2012). Contextualizing context for synthetic biology–identifying causes of failure of synthetic biological systems. Biotechnol. J..

[26] Lou C, Stanton B, Chen Y-J, Munsky B, Voigt CA (2012). Ribozyme-based insulator parts buffer synthetic circuits from genetic context. Nat. Biotechnol..

[27] Kosuri S, Goodman DB, Cambray G, Mutalik VK, Gao Y, Arkin AP, Endy D, Church GM (2013). Composability of regulatory sequences controlling transcription and translation in *Escherichia coli*. Proc. Natl Acad. Sci. U.S.A..

[28] Takahashi S, Furusawa H, Ueda T, Okahata Y (2013). Translation enhancer improves the ribosome liberation from translation initiation. J. Am. Chem. Soc..

[29] Hao Y, Zhang ZJ, Erickson DW, Huang M, Huang Y, Li J, Hwa T, Shi H (2011). Quantifying the sequence-function relation in gene silencing by bacterial small RNAs. Proc. Natl Acad. Sci. U.S.A..

[30] Mathews DH, Sabina J, Zuker M, Turner DH (1999). Expanded sequence dependence of thermodynamic parameters improves prediction of RNA secondary structure. J. Mol. Biol..

[31] Xia T, SantaLucia J, Burkard ME, Kierzek R, Schroeder SJ, Jiao X, Cox C, Turner DH (1998). Thermodynamic parameters for an expanded nearest-neighbor model for formation of RNA duplexes with Watson-Crick base pairs. Biochemistry.

[32] Gruber AR, Lorenz R, Bernhart SH, Neuböck R, Hofacker IL (2008). The Vienna RNA websuite. Nucleic Acids Res..

[33] Chen H, Bjerknes M, Kumar R, Jay E (1994). Determination of the optimal aligned spacing between the Shine–Dalgarno sequence and the translation initiation codon of *Escherichia coli* mRNAs. Nucleic Acids Res..

[34] Pertzev AV, Nicholson AW (2006). Characterization of RNA sequence determinants and antideterminants of processing reactivity for a minimal substrate of *Escherichia coli* ribonuclease III. Nucleic Acids Res..

[35] Keseler IM, Collado-Vides J, Santos-Zavaleta A, Peralta-Gil M, Gama-Castro S, Muñiz-Rascado L, Bonavides-Martinez C, Paley S, Krummenacker M, Altman T (2011). EcoCyc: a comprehensive database of *Escherichia coli* biology. Nucleic Acids Res..

[36] Mutalik VK, Qi L, Guimaraes JC, Lucks JB, Arkin AP (2012). Rationally designed families of orthogonal RNA regulators of translation. Nat. Chem. Biol..

[37] Rodrigo G, Landrain TE, Jaramillo A (2012). De novo automated design of small RNA circuits for engineering synthetic riboregulation in living cells. Proc. Natl Acad. Sci. U.S.A..

[38] Callura JM, Cantor CR, Collins JJ (2012). Genetic switchboard for synthetic biology applications. Proc. Natl Acad. Sci. U.S.A..

[39] Sinha J, Reyes SJ, Gallivan JP (2010). Reprogramming bacteria to seek and destroy an herbicide. Nat. Chem. Biol..

[40] Anthony PC, Perez CF, García-García C, Block SM (2012). Folding energy landscape of the thiamine pyrophosphate riboswitch aptamer. Proc. Natl Acad. Sci. U.S.A..

[41] Perdrizet GA, Artsimovitch I, Furman R, Sosnick TR, Pan T (2012). Transcriptional pausing coordinates folding of the aptamer domain and the expression platform of a riboswitch. Proc. Natl Acad. Sci. U.S.A..

[42] Lynch SA, Desai SK, Sajja HK, Gallivan JP (2007). A high-throughput screen for synthetic riboswitches reveals mechanistic insights into their function. Chem. Biol..

[43] Caron M-P, Bastet L, Lussier A, Simoneau-Roy M, Massé E, Lafontaine DA (2012). Dual-acting riboswitch control of translation initiation and mRNA decay. Proc. Natl Acad. Sci. U.S.A..

[44] Updegrove T, Wilf N, Sun X, Wartell RM (2008). Effect of Hfq on RprA−rpoS mRNA pairing: Hfq−RNA binding and the influence of the 5′ rpoS mRNA leader region. Biochemistry.

[45] Sacerdot C, Caillet J, Graffe M, Eyermann F, Ehresmann B, Ehresmann C, Springer M, Romby P (1998). The *Escherichia coli* threonyl-tRNA synthetase gene contains a split ribosomal binding site interrupted by a hairpin structure that is essential for autoregulation. Mol. Microbiol..

[46] Gabashvili IS, Agrawal RK, Grassucci R, Frank J (1999). Structure and structural variations of the *Escherichia coli* 30 S ribosomal subunit as revealed by three-dimensional cryo-electron microscopy. J. Mol. Biol..

[47] Clemons WM, May JL, Wimberly BT, McCutcheon JP, Capel MS, Ramakrishnan V (1999). Structure of a bacterial 30S ribosomal subunit at 5.5 Å resolution. Nature.

[48] Pioletti M, Schlünzen F, Harms J, Zarivach R, Glühmann M, Avila H, Bashan A, Bartels H, Auerbach T, Jacobi C (2001). Crystal structures of complexes of the small ribosomal subunit with tetracycline, edeine and IF3. EMBO J..

[49] Williamson JR (2000). Induced fit in RNA–protein recognition. Nat. Struct. Mol. Biol..

[50] Fiore JL, Holmstrom ED, Nesbitt DJ (2012). Entropic origin of Mg2+-facilitated RNA folding. Proc. Natl Acad. Sci. U.S.A..

[51] Mills JB, Vacano E, Hagerman PJ (1999). Flexibility of single-stranded DNA: use of gapped duplex helices to determine the persistence lengths of poly (dT) and poly (dA). J. Mol. Biol..

[52] Egbert RG, Klavins E (2012). Fine-tuning gene networks using simple sequence repeats. Proc. Natl Acad. Sci. U.S.A..

