# Supplementary material for: Translation rate is controlled by coupled trade-offs between site accessibility, selective RNA unfolding and sliding at upstream standby sites
Source: Nucleic Acids Res. 2013 Nov 14;42(4):2646–59. doi: 10.1093/nar/gkt1139 (PMC3936740; doi:10.1093/nar/gkt1139)
Supplement: Supplementary Data [file supp_42_4_2646__index.html]

Translation rate is controlled by coupled trade-offs between site accessibility, selective RNA unfolding and sliding at upstream standby sites — Translation rate is controlled by coupled trade-offs between site accessibility, selective RNA unfolding and sliding at upstream standby sites — Supplementary Data 

# Translation rate is controlled by coupled trade-offs between site accessibility, selective RNA unfolding and sliding at upstream standby sites

## Supplementary Data

files

**Files in this Data Supplement:**

- Supplementary Data - pdf file
- Supplementary Data - xls file
