# Supplementary material for: Translation rate is controlled by coupled trade-offs between site accessibility, selective RNA unfolding and sliding at upstream standby sites
Source: Nucleic Acids Res. 2013 Nov 14;42(4):2646–59. doi: 10.1093/nar/gkt1139 (PMC3936740; doi:10.1093/nar/gkt1139)
Supplement: Supplementary Data [file supp_gkt1139_nar-02492-r-2013-File009.pdf]

Supplementary Text

Translation Rate is Controlled by Coupled Trade-offs Between Site Accessibility, Selective RNA Unfolding, and Sliding at Upstream Standby Sites

Amin Espah Borujeni[1], Anirudh S Channarasappa[1], and Howard M Salis[1,2,]*

[1]Department of Chemical Engineering, Penn State University, University Park, PA, 16802, USA

[2]Department of Biological Engineering, Penn State University, University Park, PA, 16802, USA

*To whom correspondence should be addressed. Tel: 814-865-1931. Email: salis@psu.edu

Table of Supplementary Figures:

Figure S1. Translation Initiation Begins with Ribosomal Platform's Binding to Structured 5' UTRs

Figure S2. Effect of Structured 5' UTR on mRNA Stability and Translation Initiation Rate

Figure S3. Biophysical Model Predicts the Ribosomal Platform's Binding Free Energy Penalty to Branched Structured 5' UTRs

Figure S4. Ribosomal Platform Unfolds mRNA Structures Based on Hairpin Sequence and Energy

Figure S5. Examples of Synthetic Structured 5' UTRs Designed to Characterize the Interactions Between Ribosomal Platform and Structured 5' UTR

Figure S6. Analysis of Distribution of Error in Biophysical Model Predictions

Ribosomal Platform Interactions with Structured Standby Sites Control Translation Initiation Rate

The ribosomal platform, which makes contact with structured mRNAs at its surface, is one of the three compartments of 16S ribosomal RNA, a scaffold for 30S ribosomal subunit. Six small ribosomal proteins at the platform surface create positively charged surface and bind non-specifically to the negatively charged phosphate backbone of mRNA via electrostatic interactions. In addition, the anti-SD of 16S ribosomal RNA, which is located on the platform surface, stabilizes the mRNA through hydrogen binding (**Figure S1A**). Although about 30 nt of mRNA around the start codon has to be unfolded and fed into the mRNA channel to position the start codon at the P-site (2), the upstream 5' UTR can remain folded and in contact with the ribosomal platform surface. The efficient binding of structured mRNAs to the ribosomal platform is a prerequisite for their translation. Therefore, understanding the interactions between ribosomal platform and structured mRNAs is necessary to accurately control their translation rate.

The previously developed biophysical model of translation initiation, RBS Calculator v1.0, (14) accounts for five molecular interactions between mRNA and 30S ribosomal subunit. The total Gibbs free energy change during translation initiation, ΔG_{total}, is the quantitative measure of its rate (**Figure S1B**). The model accurately predicted the translation initiation rates of 132 mRNAs with different Shine-Dalgarno sequences, inhibitory mRNA structures, and 5' coding sequences. However, v1.0 of the biophysical model was not able to accurately calculate the ribosome's binding free energy ΔG_{total} for mRNAs that contain structures upstream of the Shine-Dalgarno sequence in the After-bound state. The predicted translation initiation rate of an example, RBS-91, was 26.25-fold greater than its measured fluorescence level (3387.86 au vs. 129.08±39.93 au on the same proportional scale), equivalent to neglecting a 7.26 kcal/mol binding free energy penalty (**Figure S1C**). In contrast, the previous model can accurately predict the translation initiation rates of mRNAs where mRNA structures overlap with the Shine-Dalgarno sequence, but they are primarily unstructured in the After-bound state (RBS-44 example shown in **Figure S1C**).

The v1.0 of the biophysical model assumed that a small portion of standby site, which was defined as a 4 nt region upstream of the 16S rRNA binding, must be unstructured and that the energy to

unfold this region would lower the translation initiation rate. However, based on this and other data, it was clear that this assumption was false, and that the interactions controlling the ribosomal platform's ability to bind standby sites were not correctly modeled. Therefore, it was important to develop a new biophysical model to accurately calculate the ribosomal platform's interactions with standby sites, denoted by $\Delta G_{standby}$.

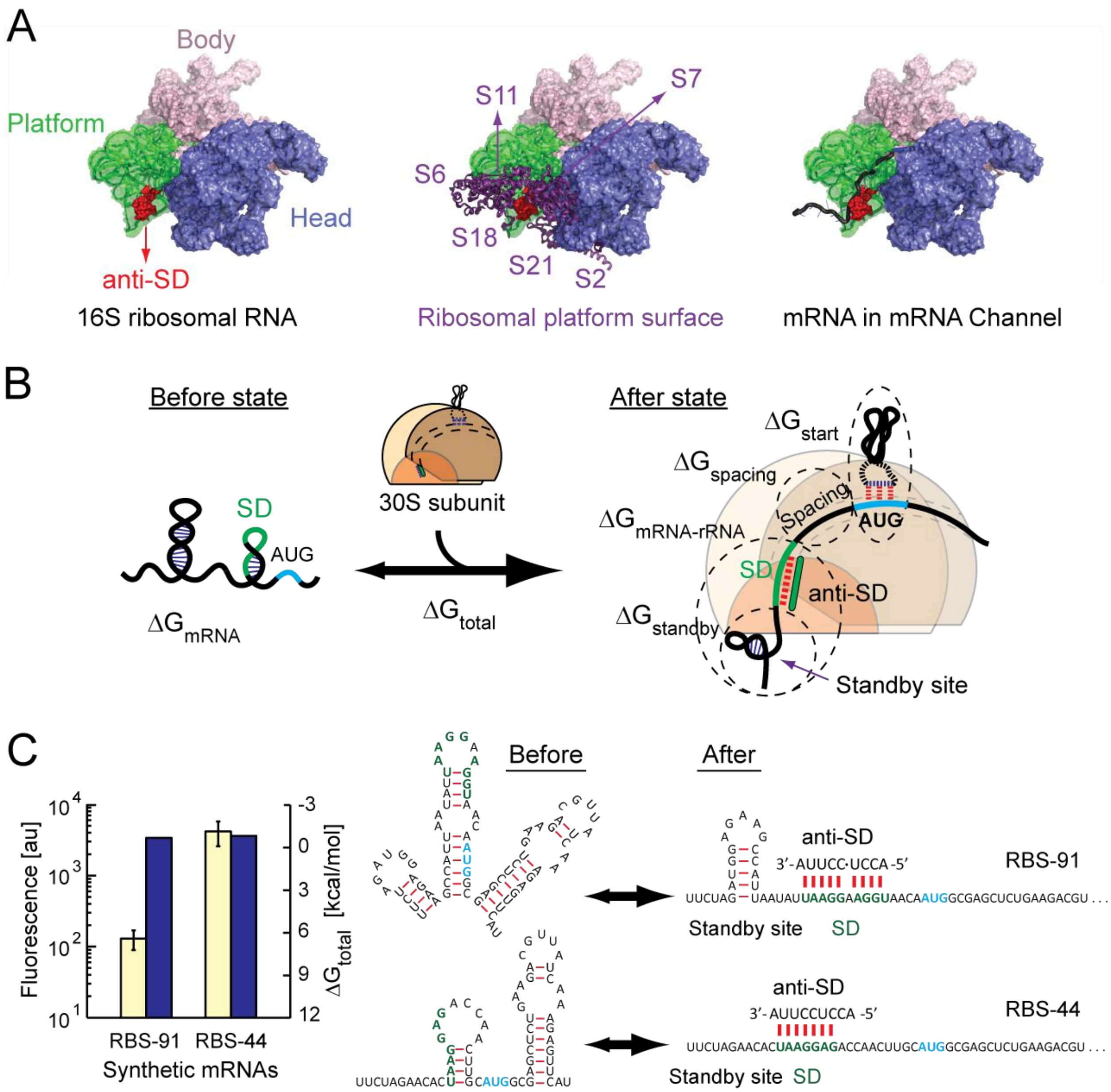

Figure S1. Translation Initiation Begins with Ribosomal Platform's Binding to Structured 5' UTRs

(A) The 16S ribosomal RNA of 30S subunit from wild type *E. coli* is shown with its three compartments. Six small ribosomal proteins create the ribosomal platform surface to bind to mRNAs. mRNA (black color) passes across this surface and enters the mRNA channel of 16S rRNA (PDB entry 3J0U). (B) Total Gibbs free energy change during translation initiation, ΔG_{total}, controls the translation initiation rate. (C) Previous model (14) inaccurately calculated ΔG_{total} of 5' UTRs with structured standby sites. Measured fluorescence (light yellow bar) is compared to predicted translation initiation rate (blue bar). RBS-91 and RBS-44 are from Ref. 14.

The Relationship Between Ribosomal Platform Binding and mRNA Stability

We measured the fluorescent protein expression levels and mRNA levels from structured mRNAs with standby site modules containing either short, medium, and long distal or proximal binding site lengths, and compared these measurements to those from an unstructured mRNA (see **Methods** in main text). In all cases, the structured mRNAs contain a hairpin with a 15 nt height. We observed a 9.6-fold reduction in fluorescence for a short proximal binding site (P = 4 nt), compared to 5.4-fold reduction in mRNA level (**Figure S2.B**). Similarly, we observed a 10.1-fold reduction in fluorescence for a short distal binding site (D = 5 nt), compared to a 3.3-fold reduction in mRNA level (**Figure S2.D**). In contrast, fluorescence levels of a medium length proximal binding site (P = 12 nt) or a medium length distal binding site (D = 12 nt) were 1.3-fold and 2.5-fold lower than an unstructured mRNA, compared to a 2.4-fold and 1.3-fold reduction in mRNA level, respectively. From this data, it appears that the hairpin itself does not significantly decrease the mRNA levels by destabilization. When shortening the proximal or distal binding site lengths, the fluorescent protein expression levels decrease more than the mRNA levels, showing that an inaccessible standby site module is primarily affecting translation rate. Though, there is likely a small amount of coupling between decreases in translation rate and decreases in mRNA stability, due to the protective effects of having ribosomes actively elongate a mRNA to prevent the binding of RNAses.

Long proximal (P = 24 nt) and distal (D = 20 nt) binding sites caused further reduction in fluorescence level (3.3- and 4.2- folds), comparable to the reduction in their mRNA levels (5.8- and 2.7-folds, respectively) (**Figure S2B** and **Figure S2D**). These reductions in fluorescence level and mRNA levels cannot be explained by the proposed biophysical model of the ribosomal platform's interactions with standby sites. Instead, these changes are likely due to increased RNAse binding to the long,

unstructured proximal or distal binding sites, causing increased mRNA degradation and lower mRNA levels. The error in the biophysical model predictions is larger when long proximal or distal binding sites (greater than 15 nt) are utilized in mRNAs, due to the confounding effects of increased mRNA degradation (see **Figure S6**).

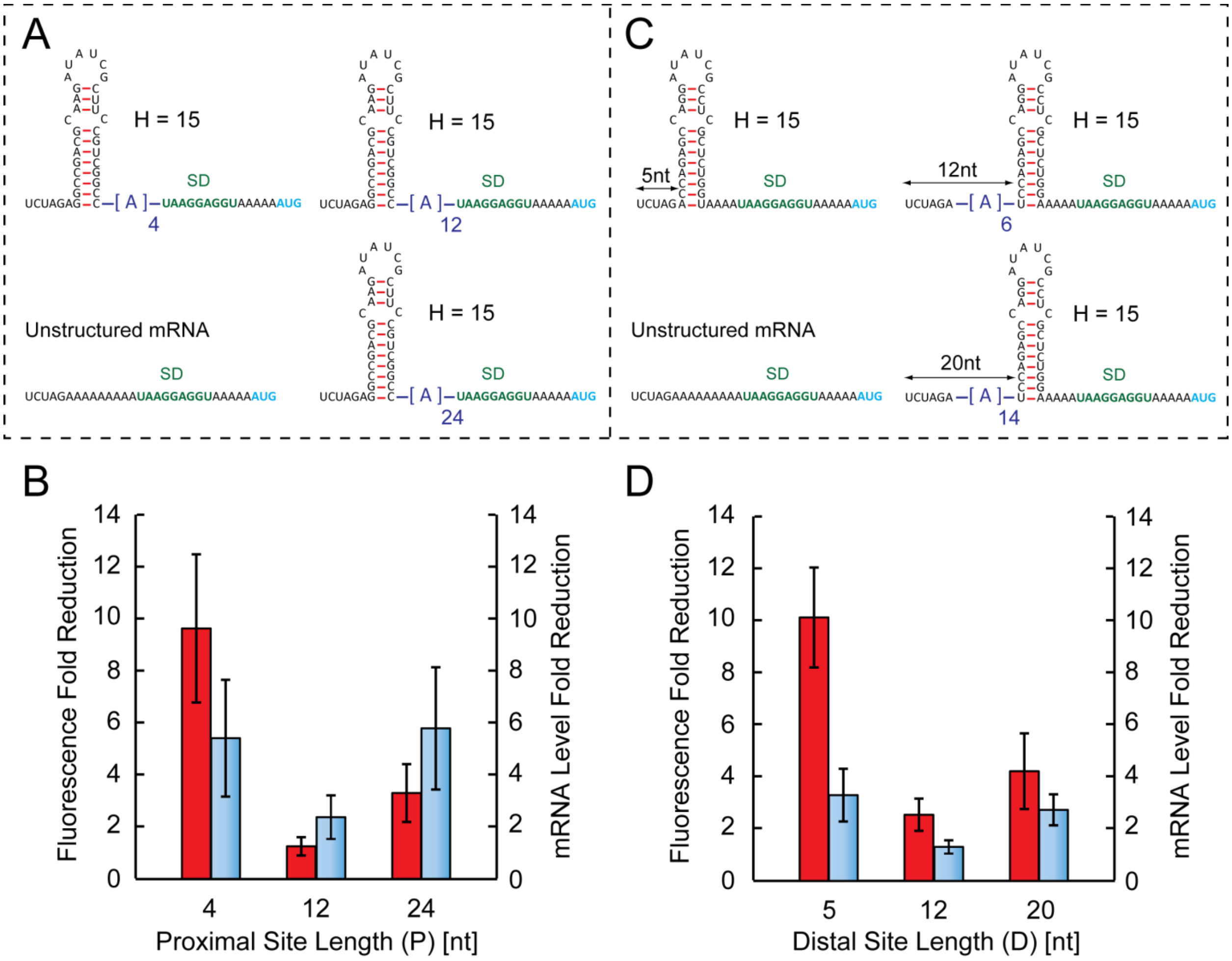

Figure S2. Effect of Structured 5' UTR on mRNA Stability and Translation Initiation Rate

(A and B) Three structured 5' UTRs with constant distal binding site length and hairpin height (D = 6 nt and H = 15 nt), but different proximal binding site length (P = 4, 12, and 24 nt) were used to examine the effect of *proximal* binding site length on mRNA instability and translation initiation rate. The fluorescence fold reduction (dark red bar), with respect to an unstructured 5' UTR, is compared to the mRNA level fold reduction (gradient light blue bar). (C and D) Three structured 5' UTRs with constant proximal binding site length and hairpin height (P = 4 nt and H = 15 nt) but different distal binding site length (D = 5, 12, and 20 nt) were used to test the effect of *distal* binding site length on mRNA instability and translation initiation rate. (D) The fluorescence fold reduction (dark red bar) is compared to the

mRNA level fold reduction (gradient light blue bar). Fluorescence data are the result of three measurements in one day and mRNA level data are the result of two measurements in separate days.

Branched Structured 5' UTRs Control the Ribosomal Platform's Binding According to their Effective Hairpin Height

Branched mRNA structures can control the ribosomal platform's distortion energy penalty differently than the normal mRNA structures. Because of the three dimensional orientations of the hairpin stems, we propose that a two-branched structure (**Figure S3A**) can bend over the neighboring proximal or distal single stranded RNA regions in three different ways (**Figure S3B**). It can bend over the single stranded RNA using its base hairpin stem together with one of its branched hairpin stems (stem I or II) (top and bottom configurations in **Figure S3B**) or only by using the base hairpin stem (middle configuration in **Figure S3B**). At each configuration, a different length of single stranded RNA is sequestered, resulting in different available RNA surface areas (A_S). We assume that the probability of each configuration is equal. Therefore, the effective A_S for a two-branched structure is the average of three available RNA surface areas, according to formula: $A_S = 15 + P + D - H_{avg}$, where the average branched hairpin height, H_{avg}, is calculated as $H_{avg} = \frac{H_1+H_2+H_3}{3}$.

In order to calculate H_1, H_2, H_3, the branched hairpin stems (stems I or II) are counted as an extra nucleotide on the multi-branched loop (**Figure S3CD**). In general, because H_2 is less than H_1 and H_3, the average branched hairpin height is shorter than the maximum hairpin height. This leads to a larger available RNA surface area and therefore lower ribosomal platform's distortion energy penalty. Importantly, this proposed formulation of average branched hairpin height can be expanded to higher number of branched stems. For example, the average branched hairpin height of a three-branched structure is calculated as $H_{avg} = \frac{H_1+H_2+H_3+H_4}{4}$, where calculation of individual hairpin heights follows the same procedure shown in **Figure S3CD**. We critically tested this proposed formulation, by predicting $\Delta G_{distortion}$ of five two-branched and one three-branched structures, with and without average branched hairpin height calculation. The average branched hairpin height calculation led to an improvement in

accuracy of predicted $\Delta G_{\text{distortion}}$ (average error is 0.38 kcal/mol with average branched hairpin height compared to 1.40 kcal/mol with maximum hairpin height (H = 15 nt); p-value is 0.017) (**Figure S3EF**).

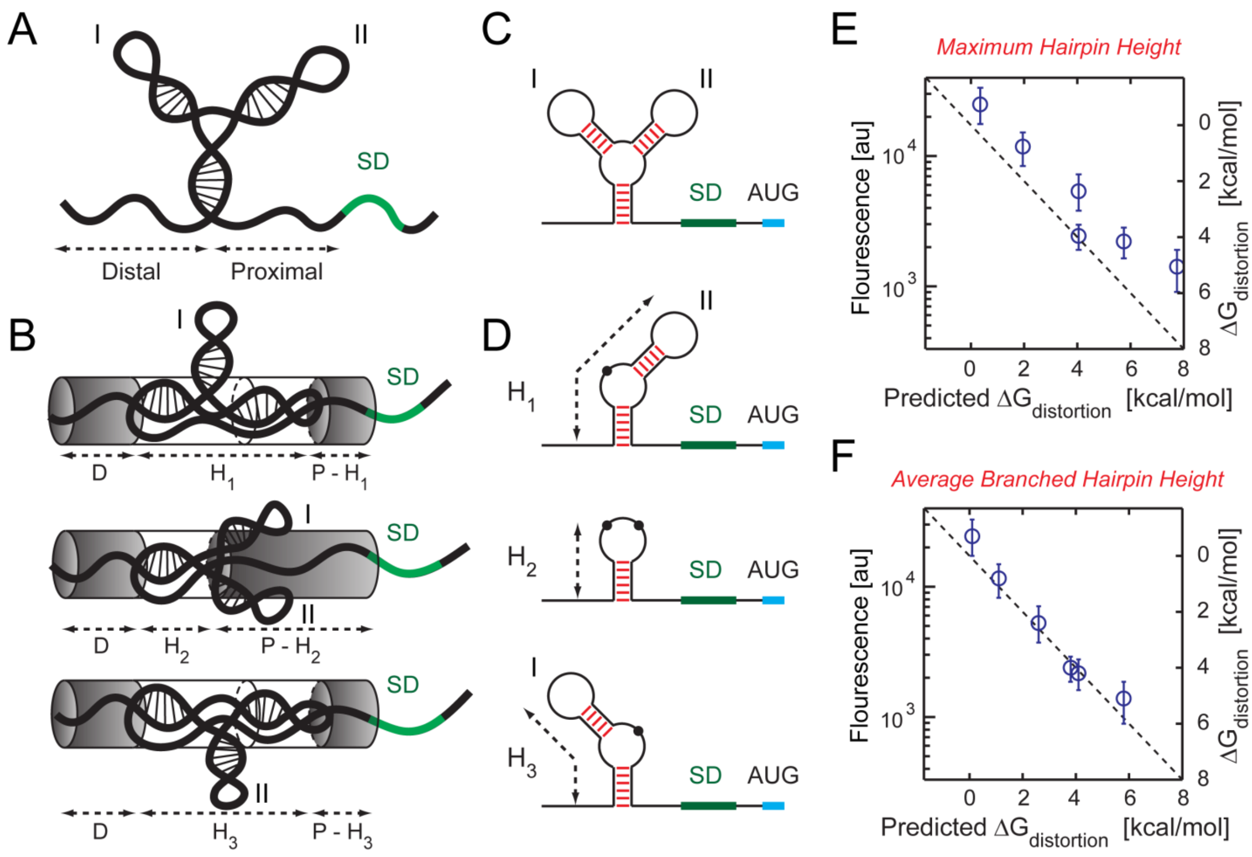

Figure S3. Biophysical Model Predicts the Ribosomal Platform's Binding Free Energy Penalty to Branched Structured 5' UTRs

(A and B) A standby site module with a two-branched hairpin is shown. The two-branched hairpin can bend over adjacent single stranded region in three different orientations. The available RNA surface area (shaded) depends on the hairpin's bending orientation. (C and D) Schematic representation of a two-branched structure is shown. The effective hairpin height in each orientation (H_1, H_2, H_3) is depicted. A hairpin stem on the multi-branched loop is counted as an extra nucleotide (black dot). (E and F) Predicted $\Delta G_{\text{distortion}}$ using maximum hairpin heights (H = 15 nt) of five two-branched structures and one three-branched structure (**Supplementary Data**) are compared to predictions using average branched hairpin height (average errors are 1.40 and 0.38 kcal/mol, respectively; p-value is 0.017). In parts EF, $\Delta G_{\text{distortion}}$ numbers shown in secondary y-axis are calculated using **Equation 3** and are

directly related to the data according to **Equation 4**. The diagonal dashed line is also the predicted translation rate according to **Equation 4**, where $\Delta\Delta G_{other}$ is zero kcal/mol.

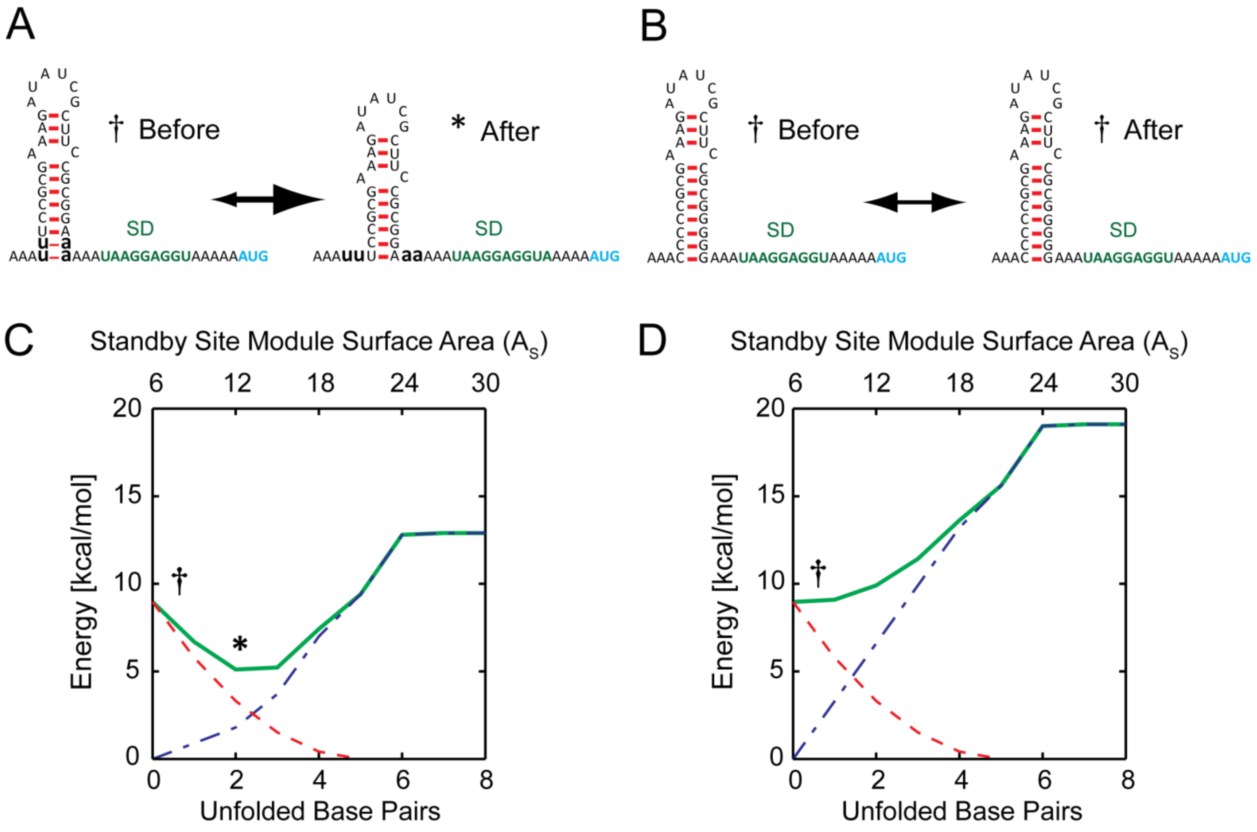

Figure S4. Ribosomal Platform Unfolds mRNA Structures Based on Hairpin Sequence and Energy

(A and C) The hypothetical hairpin with A:U rich closing base pairs shows a moderate slope for unfolding energy landscape. Two closing base pairs (bolded lower case) are predicted to be unfolded by the ribosomal platform in order to reach the minimum binding free energy penalty (5.11 kcal/mol). (B and D) The hypothetical hairpin with G:C rich closing base pairs has a steep unfolding energy landscape. No closing base pair is predicted to be unfolded. The fully folded state (A_S = 6) has the minimum binding free energy penalty (8.96 kcal/mol). $\Delta G_{distortion}$ is dashed red line, $\Delta G_{unfolding}$ is dotted-dashed blue line, and $\Delta G_{standby}$ is solid green line.

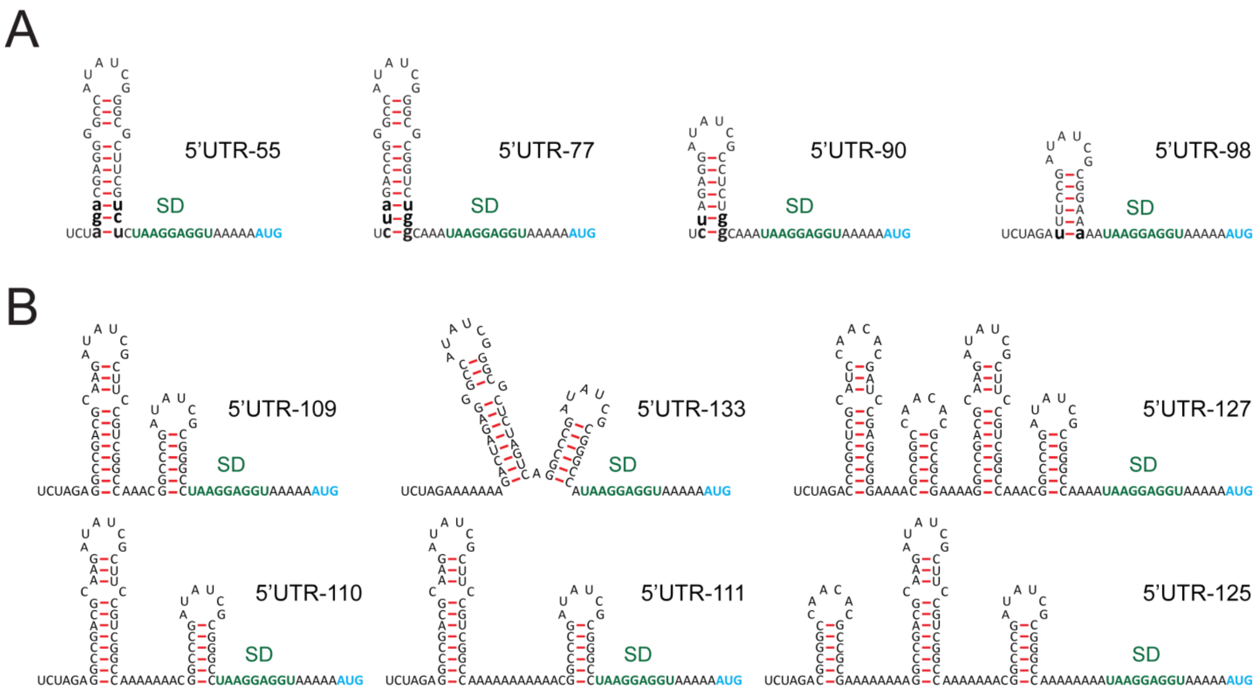

Figure S5. Examples of Synthetic Structured 5' UTRs Designed to Characterize the Interactions Between Ribosomal Platform and Structured 5' UTR

(A) Designed structured 5' UTRs with diverse sequences demonstrate the coupled trade-offs between ribosomal distortion and RNA unfolding. The predicted unpaired bases are shown in bold lower cases. (B) Synthetic 5' UTRs with diverse structures were designed to examine the ability of biophysical model in predicting the ribosomal platform's binding free energy penalty to the structured mRNAs. Sequence, size, and folding energy of all mRNA structures are listed in **Supplementary Data**.

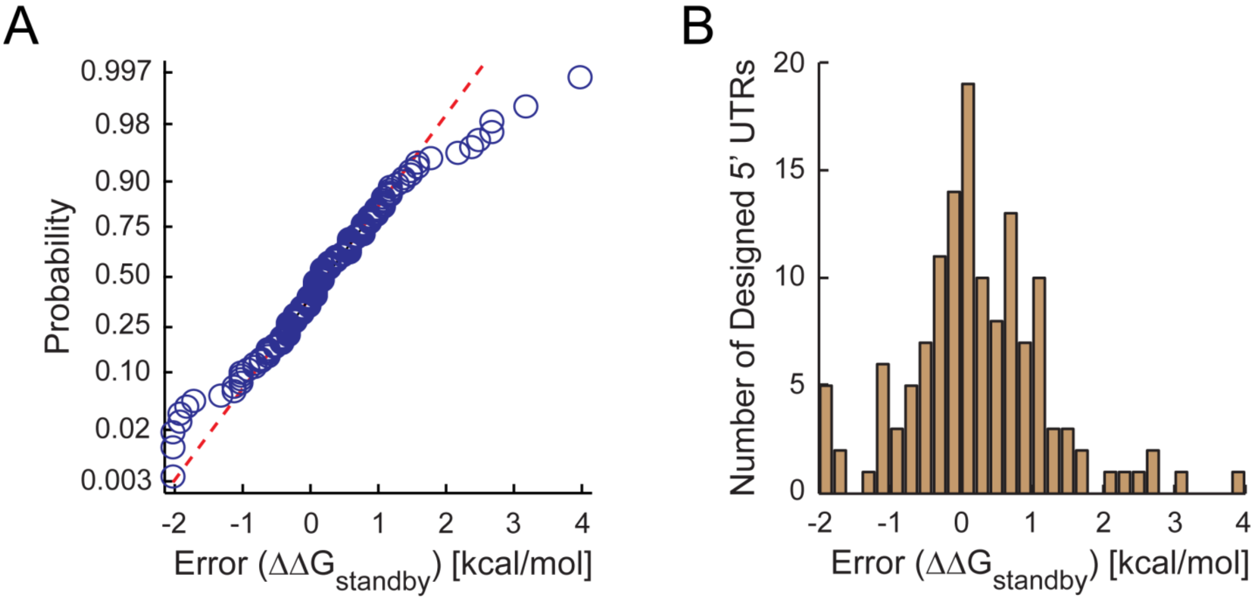

Figure S6. Analysis of Distribution of Error in Biophysical Model Predictions

(A) The probability plot for normal distribution of errors in predicted $\Delta G_{\mathrm{standby}}$ (Anderson-Darling test with 95% confidence interval) shows that the error is normally distributed across 90% of the designed 5' UTRs. However, 13 designed 5' UTRs show deviation from normality, indicating some systematic errors in model prediction. Six of these designed 5' UTRs are under-predicted by the biophysical model (shown by red color background in the **Supplementary Data**), which are mostly affected by mRNA instability (See **Figure S2**). Others are over-predicted by the biophysical model (shown by blue color background in the **Supplementary Data**) (see also **Discussion**). The red-dashed line joins the first and third quartiles of sample population. (B) A histogram shows that the biophysical model is capable of predicting $\Delta G_{\mathrm{standby}}$ for most of designed 5' UTRs. $\Delta\Delta G_{\mathrm{standby}}$= measured $\Delta G_{\mathrm{standby}}$- predicted $\Delta G_{\mathrm{standby}}$.
